# Emotion dysregulation links pathological eating styles and psychopathological traits in bariatric surgery candidates

**DOI:** 10.3389/fpsyt.2024.1369720

**Published:** 2024-03-28

**Authors:** Arianna Belloli, Luigi F. Saccaro, Paola Landi, Milena Spera, Marco Antonio Zappa, Bernardo Dell’Osso, Grazia Rutigliano

**Affiliations:** ^1^ Department of Psychiatry, Azienda Socio Sanitaria Territoriale (ASST) Fatebenefratelli-Sacco, Milan, Italy; ^2^ Department of Psychology, Sigmund Freud University, Milan, Italy; ^3^ Department of Psychiatry, Faculty of Medicine, University of Geneva, Geneva, Switzerland; ^4^ Department of Psychiatry, Geneva University Hospital, Geneva, Switzerland; ^5^ Department of General Surgery, Azienda Socio Sanitaria Territoriale (ASST) Fatebenefratelli-Sacco, Milan, Italy; ^6^ Institute of Clinical Sciences, Faculty of Medicine, Imperial College London, London, United Kingdom

**Keywords:** obesity, bariatric surgery, eating style, emotion regulation, network analysis

## Abstract

**Objectives:**

Approximately one-third of bariatric surgery patients experience weight regain or suboptimal weight loss within five years post-surgery. Pathological eating styles and psychopathological traits (e.g., emotion dysregulation) are recognized as potential hindrances to sustain weight loss efforts and are implicated in obesity development. A comprehensive understanding of these variables and their interplays is still lacking, despite their potential significance in developing more effective clinical interventions for bariatric patients. We investigate the prevalence of and interactions between pathological eating styles and psychopathological traits in this population.

**Materials and methods:**

110 bariatric surgery candidates were characterized using the Binge Eating Scale (BES), Hamilton Depression/Anxiety Scales (HAM-D/A), Barratt Impulsiveness Scale (BIS-11), Experiences in Close Relationships (ECR), Difficulties in Emotion Regulation Scale (DERS). We analyzed these variables with multiple logistic regression analyses and network analysis.

**Results:**

Patients with pathological eating styles showed more pronounced anxiety/depressive symptoms and emotion dysregulation. Network analysis revealed strong connections between BES and DERS, with DERS also displaying robust connections with HAM-A/D and ECR scales. DERS and attention impulsivity (BIS-11-A) emerged as the strongest nodes in the network.

**Discussion:**

Our findings demonstrate the mediating role of emotion dysregulation between pathological eating styles and psychopathological traits, supporting existing literature on the association between psychopathological traits, insecure attachment styles, and pathological eating behaviors. This research emphasizes the significance of emotion regulation in the complex network of variables contributing to obesity, and its potential impact on bariatric surgery outcomes. Interventions focusing on emotion regulation may thus lead to improved clinical outcomes for bariatric patients.

## 1 Introduction

Obesity is a metabolic and neuroendocrine condition characterized by an excessive accumulation or abnormal distribution of body fat, related to health complications. The etiology of obesity is complex and multifactorial, characterized by the interplay between biological and psychosocial factors (1). Bariatric surgery refers to a variety of inner gastrointestinal surgical techniques that were first intended to help patients who were morbidly obese lose weight. However, the advantages of bariatric surgery go far beyond weight loss and include a significant improvement in type 2 diabetes, hypertension, dyslipidemia, and a decrease in overall mortality. Bariatric surgeries, performed laparoscopically, can be classified into: restrictive (gastric banding and sleeve gastrectomy), malabsorptive (biliopancreatic diversion and mini gastric bypass) and mixed. Restrictive interventions are based on gastric volume reduction, malabsorptive ones are aimed at reducing the size of the stomach by modifying its digestive process, and mixed interventions, such as gastric bypass, reduce the volume of the stomach pouch and the surface area intestinal destined for absorption. Bariatric surgery has significant evidence of efficacy and safety (2) and it currently represents the best intervention for obesity in terms of weight reduction, complication control, and improvement of the quality of life (3).

A thorough psychological assessment, with particular attention to pathological eating styles, can be a useful clinical tool for managing treatment before and after bariatric surgery (4) In the present study, conducted on an Italian sample, we referred to the following taxonomy of eating styles proposed by the Italian Society of Surgery of Obesity and Metabolic Diseases: emotional eating, binge eating, qualitative eating, quantitative eating, gorging, and snacking (3). Details about the main pathological eating styles are provided in the **Supplementary Material**.

Preoperative psychological assessment is critical for bariatric surgery candidates, because it may help stratify patients based on risk of weight regain after surgery and other post-surgical outcomes (5). For an effective treatment for obesity, it is essential to consider and, ideally to treat, the underlying psychopathology maintaining abnormal eating styles (6). The psychological profile of obese patients is often characterized by anxious and depressive symptoms, impulsivity, insecure and poor attachment quality, low self-esteem, and body dissatisfaction (7). The presence of these psychopathological traits could lead to food consumption as a dysfunctional coping strategy in response to negative emotions, in absence of effective emotion regulation strategies, resulting in development of obesity (8). During the eligibility evaluation for bariatric surgery, emotion regulation and emotion recognition are seldom assessed, although neurobiological processes concerning self-regulation, including control over eating behaviors, are strongly influenced by emotions (9). Emotion regulation can be defined as the “attempt to influence which emotions we have, when we have them, and how these emotions are experienced or expressed” (10). If emotion regulation fails, self-regulation in other areas, like control over eating behaviors, may fail as well (11), hence the importance of emotion dysregulation in pathological eating behaviors, besides in other psychiatric disorders (12). Impulsiveness is also a common trait in patients who present dysregulation in food intake, especially those diagnosed with Binge Eating Disorder, whose psychological profile is characterized by significant impulsivity levels (13). Therefore, the exploration of emotion regulation and impulsivity is crucial in obese patients seeking bariatric surgery (14). Increasing evidence suggests that insecure attachment plays a crucial role in the development of obesity (15). Research indicates that insecure attachment, either anxious and avoidant, is positively associated with emotion dysregulation and can predict disordered eating behaviors, particularly in women (16, 17). In this regard, attachment history could be a key factor to consider in the prevention and treatment of overweight and obesity. Children whose caregiver is unreliable and unresponsive to their needs, may turn to food as a coping mechanism, in absence of alternative functional strategies to manage their emotions. This attitude frequently persists even into adulthood (18). In this regard we hypothesized that the main psychological factors which maintain pathological eating behaviors are emotion dysregulation, impulsivity, and insecure attachment styles.

Nevertheless, it is currently unclear how emotion dysregulation, impulsivity, and insecure attachment styles contribute to the development and maintenance of eating behaviors that can lead to obesity. Understanding these mechanisms is therefore relevant in order to improve bariatric surgery outcomes. To fill this gap, we assessed psychopathological traits and eating behaviors in a sample of bariatric surgery candidates,and applied a network analysis to clarify the interplay between psychopathological traits and eating behaviors. A network statistical approach is essential for investigating the complex interplay between emotion regulation difficulties, pathological eating behaviors, and psychopathological traits in individuals who are candidates for bariatric surgery, as it enables the identification of key nodes within the network that can be targeted for intervention, leading to more effective treatments. A network analysis specifically allows to identify the variables within the psychological profile to be addressed for intervention to prevent weight regain, providing a framework that may help to improve the long-term outcomes of bariatric surgery (19).

## 2 Materials and methods

### 2.1 Population

110 consecutive subjects (mean age: 45.04 years, standard deviation: 10.44 years, 77% females) were prospectively recruited from the multidisciplinary obesity surgery center of the Fatebenefratelli Sacco Hospital in Milan, between January 2019 and December 2021. Subjects were included if they were scheduled for bariatric surgery, according to SICOb (the Italian Society of Surgery for Obesity and Metabolic Diseases) criteria for bariatric surgery (detailed in the **Supplementary Material**). They were excluded if they were younger than 18, if they did not speak Italian, or if they did not consent. They underwent a clinical interview to exclude any mental disorders comorbidity, to measure psychopathological traits, and to assess eating behavioral styles. Thus, for instance, patients were included if they presented binge eating style, but not if they presented the full-blown Binge Eating Disorder. Patients signed an informed consent, and their identity was protected. The study was conducted in accordance with the Declaration of Helsinki, and approved by the Ethics Committee of Fatebenefratelli Sacco Hospital (“psycBAR36”‘, 2020).

### 2.2 Eating behavioral styles and psychopathological traits

During the evaluation of the surgery eligibility, patients were thoroughly monitored through a multi-disciplinary medical consultation (with dietologists, gastroenterologists, psychiatrists, and psychologists). In particular, a psychiatrist (PL) and psychotherapist (MS) clinically assessed and classified the eating behavior styles for each subject, through detailed clinical interviews involving specific questions to assess the presence of the following eating behavioral styles: emotional eating, binge eating, quantitative, qualitative (or external) eating, gorging, and snacking. Details about such clinical interviews are provided in the **Supplementary Material**. Various psychopathological traits were investigated using specific clinical scores for psychopathological traits. The Difficulties in Emotion Regulation Scale (DERS) was employed to assess emotion regulation difficulties. The Hamilton Depression Scale (HAM-D) and Hamilton Anxiety Scale (HAM-A) were used to measure levels of depression and anxiety, respectively. To evaluate binge eating behaviors, we used the Binge Eating Scale (BES). Impulsivity was assessed using the Barratt Impulsiveness Scale (BIS-11), which was further divided into subscales. The subscales included: Attention (BIS-11, A), Motor impulsivity/impulsiveness (BIS-11, Im), Self-control (BIS-11, Ac), Cognitive complexity (BIS-11, Cc), Perseverance (BIS-11, P), and Cognitive Instability (BIS-11, IC). Attachment styles were evaluated using the Experiences in Close Relationships - Revised (ECR-R) scale, with two subscales of attachment styles for avoidance and anxiety (Av/An).

Details about these clinical scores for psychopathological traits are provided in the **Supplementary Material**.

### 2.3 Statistical analyses

The categorical variables were described by absolute and relative frequencies, while the continuous variables by means of median and interquartile range, given their non-Gaussian distribution (evaluated with the D’Agostino and Pearson tests). Differences in the distribution of clinical scale scores between patients with and without the different dysfunctional eating habits were analyzed with independent-samples Mann-Whitney U. After quantifying missing data (**Supplementary Table 3**), we used the multiple imputation method to generate five complete datasets. Then, six separate multiple logistic regression models (including the original dataset) were run on multiply imputed datasets, with eating behavioral styles (quantity, quality, snack, gorge, binge, emotional eating) as dependent variables and gender and psychopathological traits (DERS, HAM-D/A, BES BIS-11, ECR-R) as independent variables. The results of the six analyses were then combined and we report below the results of the pooled analysis after multiple imputation. Statistical analyses were run in SPSS^©^ version 23. We adjusted the results for multiple comparisons using the Bonferroni correction for multiple comparison. In our case, the adjusted significance threshold is a p_corr_ value of 0.05/6 = 0.008.

To examine the relationship between eating disorder-specific scales (BES), psychiatric scales (HAM-D and HAM-A), and psychological/personality scales (BIS-11, ECR-R, and DERS), in our sample of candidates to bariatric surgery, we applied network analysis in R (https://www.Rproject.org/, version 3.6.3.), following the methods outlined in the tutorial paper by Epskamp et al. (2017) (20). This method mainly employs the free R packages *bootnet* and *qgraph*. In summary, a pairwise Markov random field network model was employed, allowing for the estimation of undirected edges, without implying any causal inference or direction in association, along with their accuracy. Additionally, centrality indices of nodes were estimated to determine their stability among the variables composing the network. The data used in the model were cross-sectional and did not follow a normal distribution. To address this, a “nonparanormal transformation” (20) was applied to convert the data into normally distributed form. In order to retain more robust edges in our sample and ensure interpretability, a “least absolute shrinkage and selection operator” (21) regularization technique was utilized. Furthermore, the Extended Bayesian Information Criterion (22, 23) was set to 0.5.

Lastly, robustness analyses were performed using the R package *bootnet* to ensure the stability and precision of the results. To evaluate the accuracy of the estimated edges, nonparametric bootstrapping (24) was performed, providing confidence intervals at the 95% level. To assess the role of each variable in the network, we calculated three centrality indexes (node strength, closeness, and betweenness). Node strength is a measure of how strongly a variable (which constitutes a node) is directly connected to other variables in the network (i.e. the total weighted connections that a variable has with other variables in the network) (25). Closeness centrality, i.e. the inverse of the sum of distances between the focal node and all other nodes in the network, measures how quickly information spreads from one variable to others in the network. The higher the closeness centrality value, the more central a variable is within the network, since variables with a high closeness score present the shortest distances to all others (26). Betweenness centrality quantifies the extent to which a variable acts as a bridge or mediator between other variables in the network, by measuring the number of times a node lies on the shortest path between other nodes. A higher betweenness centrality value suggests that the variable plays a crucial role in connecting different parts of the network (25).

## 3 Results

### 3.1 Characteristics of the sample

Our sample included 85 women and 25 men (total: 110, median age 47 years). The most frequent pathological eating style was the achievement of gratification with large quantity of food (n=78, 70.9%), followed by emotional eating (n=74, 68.5%), achievement of gratification based in food quality (very salty or sweet) (n=66, 60%), snacking (n=59, 53.6%), gorging (n=46, 41.8%), and binge eating (n=18, 16.4%). In 92.7% of cases (n=102) patients presented 2 or more pathological eating behaviors. 31.9% (n=30) of patients showed moderate to severe BES scores (**Table 1**).

**Table 1:** Sample characteristics, Continuous variables: Difficulties in Emotion Regulation Scale (DERS), Experiences in Close Relationships Questionnaire - Revised-Avoidance/Anxiety (ECR-Av/An), Hamilton Depression Scale (HAM-D), and Hamilton Anxiety Scale (HAM-A), Binge Eating Scale (BES), Barratt Impulsiveness Scale (BIS-11). BIS-11 is further divided into factors, including Attention (BIS-11, A), Motor impulsiveness (BIS-11, Im), Self-control (BIS-11, Ac), Cognitive complexity (BIS-11, Cc), Perseverance (BIS-11, P) and Cognitive Instability (BIS-11, IC).

Variable	Mean (SD)	Median (25%-75% percentile)
**Age**	45.04 (10.44)	47.00 (36.50-53.50)
**BES**	*13.46 (8.64)*	13.00 (6.75-20.00)
**BIS-11, A**	*12.51 (3.92)*	13.50 (9.25-16.00)
**BIS-11, Im**	*17.94 (6.23)*	19.00 (12.00-23.75)
**BIS-11, Ac**	13.18 (3.32)	14.00 (11.00-15.00)
**BIS-11, Cc**	*12.95 (2.97)*	13.00 (11.25-15.00)
**BIS-11, P**	10.08 (3.42)	11.00 (7.00-13.00)
**BIS-11, Ic**	*7.81 (2.77)*	8.00 (6.00-10.00)
**BIS-11, Total**	*74.48 (17.79)*	80.50 (57.25-88.75)
**ECR-Av-mean**	3.00 (1.00)	3.00 (2.22-3.61)
**ECR-An-mean**	*5.82 (2.28)*	5.58 (4.20-7.11)
**ECR-Av**	53.01 (18.01)	53.00 (40.00-65.00)
**ECR-An**	*54.41 (22.19)*	52.00 (38.00-67.00)
**DERS**	80.05 (23.98)	76.00 (60.75-95.00)
**HAM-D**	*10.81 (7.97)*	9.00 (5.00-16.00)
**HAM-A**	*10.88 (8.58)*	9.00 (4.00-14.00)

### 3.2 Distribution of psychopathological traits between eating styles

As detailed in **Supplementary Tables 2A–F**, we compared the distribution of psychopathological traits between patients with and without the different dysfunctional eating styles.

Higher scores in different BIS-11 domains were found among patients with gorging than in those without this eating style (BIS-11, A, attention, p=0.012; BIS-11, Im, motor impulsivity, p=0.029; BIS-11, Ac, self-control, p=0.001; BIS-11, P, perseverance, p=0.031).

Patients with binge and emotional eating showed higher scores in: BES (respectively, p<0.001 and p=0.030); ECR-R (avoidant: respectively, p=0.038 and p=0.051; anxious: respectively, p=0.019 and p=0.009); DERS (respectively, p=0.038 and p=0.056); HAM-D (p=0.012 and p=0.001) and HAM-A (p=0.012 and 0.001). Patients seeking quantitative gratification have higher scores in HAM-A (p=0.014).

The following findings survived adjustment for multiple comparisons: higher scores in BIS-11, Ac (self-control) among patients with gorging eating style (U=1220, p_corr_<0.008); higher scores in BIS-11, A (attention, U=545, p_corr_<0.008) and Im (motor impulsivity, U=518, p_corr_<0.008) among patients with qualitative eating style; higher scores in BES among patients with binge eating style (U=918, p_corr_<0.001); and higher scores in HAM-D (U=1422, p_corr_=0.001) and HAM-A (U=1421, p_corr_=0.001) in patients with emotional eating style.

### 3.3 Results of logistic regressions

The logistic regression analysis yielded several significant findings before correction for multiple comparisons (**Supplementary Table 4**). For a one-unit increase in BIS-11-Ac (self-control domain of BIS-11), the odds of gorge eating style increased by 24% (95% CI [1.003, 1.53], p=0.047). For a one-unit increase in BES, the odds of binge eating style increased by 19% (95% CI [1.024, 1.374], p=0.024). Lastly, males were almost 5 times more likely to have a quantitative eating style as compared to females (95% CI [1.053, 22.099]), while their odds of having an emotional eating style were 24% lower (95% CI [0.076, 0.748]). There were no other significant findings.

No result of the logistic regression analysis survived adjustment for multiple comparisons.

### 3.4 Results of network analysis

#### 3.4.1 General characteristics of the network structure

**Figure 1:**
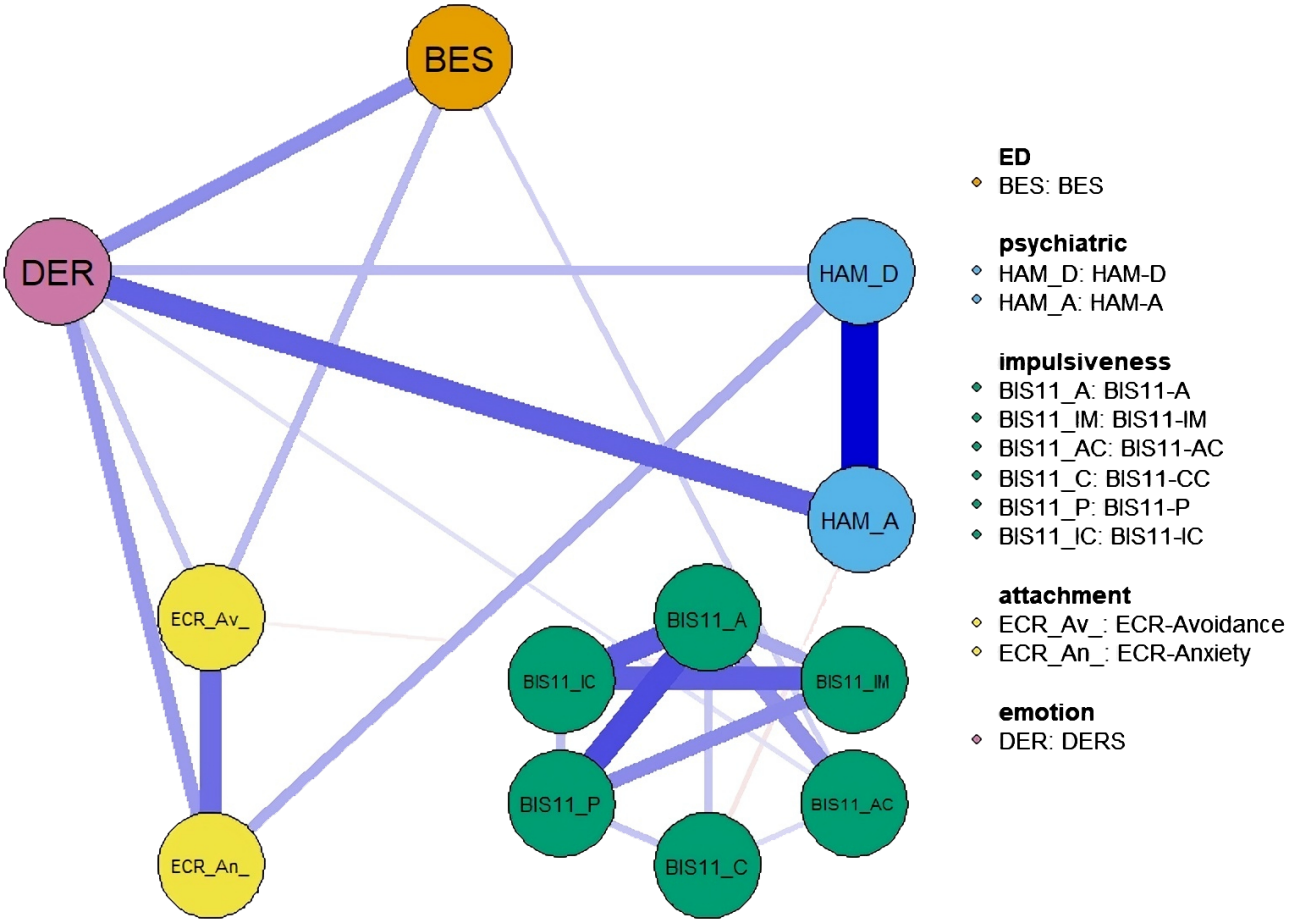
Network structure. The variables include eating disorder-specific scales (BES), psychiatric scales (HAM-D and HAM-A) and psychological and personality scales (BIS-11, ECR and DER). Item groups are differentiated by color. Edge colors represent the direction of associations (blue for positive, red for negative), and edge widths indicate the strength of these associations.

The network includes the following psychopathological traits: DERS, HAM-D/A, BES, BIS-11, ECR (**Figure 1**). The correlation matrix between the variables is shown in **Supplementary Figure 1**.

**Figure 2:**
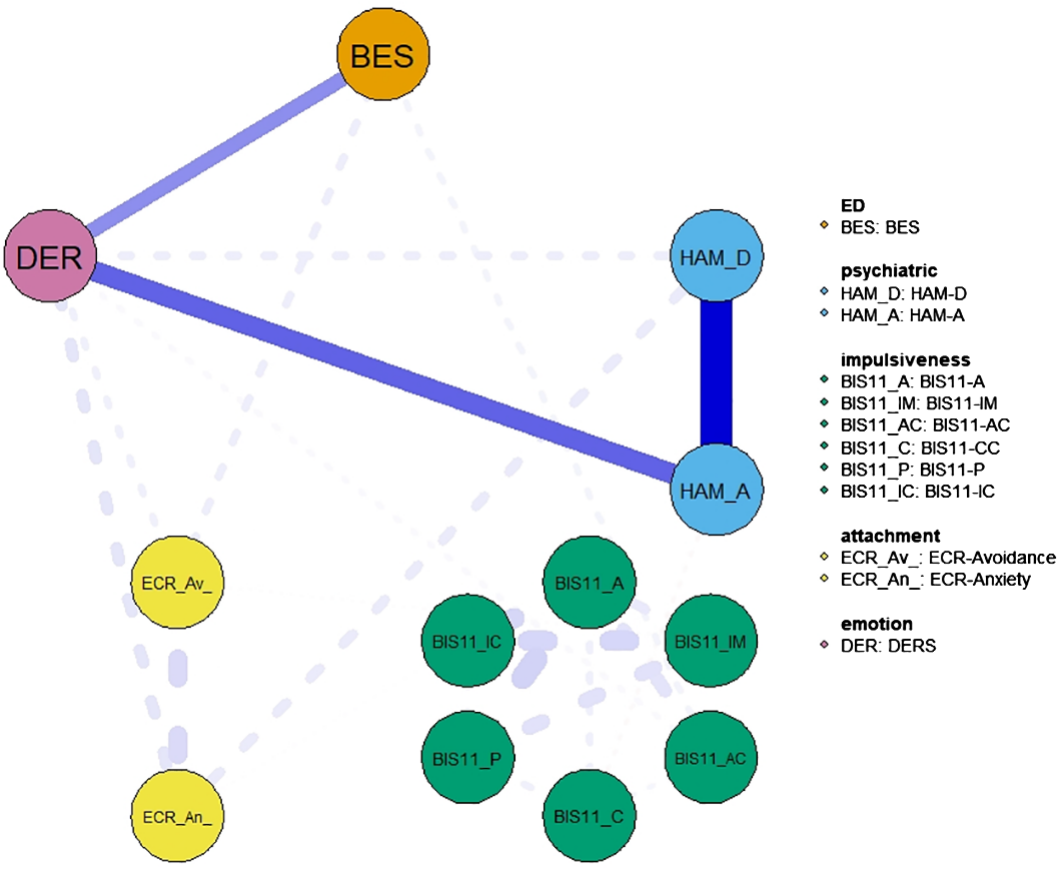
Network showing the shortest path between the BES and Hamilton depression and anxiety scales. The variables include eating disorder-specific scales (BES), psychiatric scales (HAM-D and HAM-A) and psychological and personality scales (BIS-11, ECR and DER). Item groups are differentiated by color. Edge colors represent the direction of associations (blue for positive, red for negative), and edge widths indicate the strength of these associations.

Network structure showed that all psychopathological traits were connected, either directly or indirectly, with each other through positive associations. However, psychopathological traits within the same domain were generally connected more strongly than across domains. For instance, impulsiveness scores formed an interconnected cluster that was almost isolated from the rest of the network, apart from a weak connection with BES and DERS. HAM-D and HAM-A were strongly interconnected with each other, as well as ECR-R scores. Regarding connections across domains, BES was most strongly connected with DERS, which was in turn robustly connected with HAM-A/D and ECR-R. This pattern of connectivity was confirmed by the network showing that the shortest path between the BES and HAM-A/D scales was indeed through DERS (**Figure 2**).

#### 3.4.2 Centrality indexes

**Figure 3:**
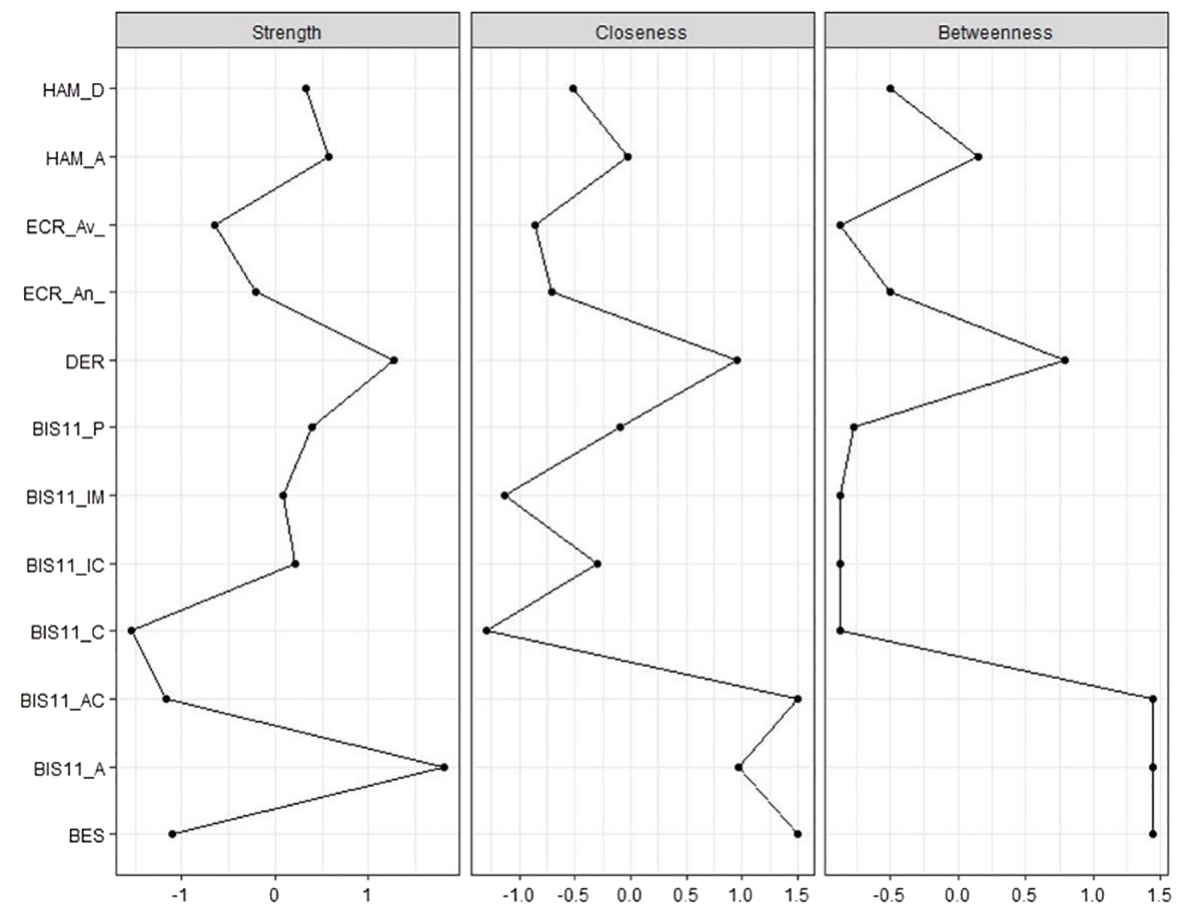
Centrality indexes (node strength, closeness and betweenness) of eating disorder-specific scales (BES), psychiatric scales (HAM-D and HAM-A) and psychological and personality scales (BIS-11, ECR and DERS) in our sample of patients candidate to bariatric surgery. standardized z-scores are shown on x-axis.

According to the centrality indexes analysis (**Figure 3**), the strongest nodes in the network were DERS and BIS-11-A (Attention impulsivity). Of note, the high node strength of BIS-11-A seems to be due to the strong connections within the BIS-11 subscores, while DERS strength derives mainly from diverse connections with the remaining variables. BIS-11-C, BIS-11-Ac, and BES had the lowest node strengths.

Closeness and betweenness were highly correlated in the network. This correlation indicates that the variables that are influential in spreading information quickly throughout the network (high closeness centrality) are also likely to be important in facilitating communication and interactions between different nodes (high betweenness centrality).

The variables with highest closeness and betweenness were DERS, BIS-11-C, BIS-11-Ac, and BES.

These variables act therefore as critical intermediaries, linking different clusters or groups within the network and enabling the flow of information between them.

Further details on stability of centrality indices (**Supplementary Figure 3**), as well as networks showing the shortest paths between BES and other variables are available in **Supplementary Information** (**Supplementary Figures 4**-**6**).

#### 3.4.3 Robustness analysis

We assessed the accuracy and stability of edge-weights through robustness analyses (**Supplementary Figure 2**). These analyses employed bootstrapped confidence intervals for estimated edge-weights within the network encompassing ED-specific scales (BES), psychiatric scales (HAM-D and HAM-A), and psychological and personality scales (BIS-11, ECR, and DERS). These robustness analyses revealed the presence of relatively wide bootstrapped confidence intervals, suggesting a need for caution, especially when interpreting weaker edges. However, it’s noteworthy that the sample values predominantly fall within the bootstrapped confidence intervals, and the bootstrap mean values generally align well with the sample values. These outcomes collectively indicate reasonably accurate estimations and offer results that can be sensibly interpreted.

The average correlations between centrality indices (strength and betweenness) were examined across networks generated by dropping varying proportions of the data through 2500 iterations. The findings indicate that the central stability coefficient, which signifies the maximum drop proportions necessary to maintain a correlation of 0.7 in at least 95% of the sample, was 0.365 for strength and 0 for betweenness (**Supplementary Figure 3**). It is important to note that networks with dependable centrality should exhibit a stability coefficient equal to or greater than 0.25, ideally surpassing 0.5 for centrality estimates (27). Consequently, particular caution is warranted when interpreting these results and the centrality of betweenness, which may lack stability.

## 4 Discussion

The purpose of the present study is to provide novel evidence on the association between pathological eating styles and psychopathological traits, employing network analysis in a sample of bariatric surgery candidates. The findings of the current study highlight the importance of considering these interconnected factors when assessing and treating individuals with pathological eating behaviors and, specifically, those who are candidates for bariatric surgery.

Finally, we found that subjects with binge eating and emotional eating had a higher psychopathological burden with higher scores on different scales (DERS, HAM-D, HAM-A, BES, and ECR-R). More in detail, our network analysis shows that emotion regulation difficulties have a pivotal role in linking the positive association between anxious attachment style and binge eating, as well as the association between binge eating and symptoms of anxiety and depression. This is in line with existing literature, which shows that emotion dysregulation may be a crucial underlying mechanism linking insecure attachment to emotional eating and other dysfunctional eating styles in bariatric patients (28). In fact, a meta-analytic review (29) highlights how an insecure attachment of the anxious type is significantly associated with dysfunctional eating behaviors such as: binges, bulimic symptoms, consumption of unhealthy food, and emotional eating, as compared to a safe attachment. For these individuals food becomes a self-regulation and self-assurance tool, highlighting the link between emotion regulation and food seeking. These results therefore suggest that psychological interventions aimed at reducing emotion dysregulation may help decrease dysfunctional eating behaviors in bariatric surgery candidates (30). In our study, impulsivity emerged as the other significant factor in the pathophysiology of dysfunctional eating behaviors, specifically gorging. In fact, patients who engaged in gorging had higher scores in the self-control domain of the Impulsiveness Scale. The relationship between impulsivity and pathological eating may be also linked by emotion dysregulation (14).

Our study shows that network analysis represents a promising approach for the exploration of the mechanisms underlying the psychological profile of bariatric surgery candidates. Although little literature (31) is currently available on network analysis applied to bariatric surgery, there is novel evidence on the use of network analysis in the field of eating disorders (EDs) (32–38). Solmi and colleagues applied network analysis to assess ED-specific symptoms, psychiatric symptoms, and general clinical variables on a sample of patients diagnosed with EDs. Their results suggested the central role of drive for thinness, interpersonal functioning, ineffectiveness, interoceptive awareness, and affective symptoms in EDs (39). Although this study does not specifically treat bariatric patients, it stresses like our study how a network approach may highlight a maintaining psychopathologic loop which appears to involve, but transcends, dimensions peculiar to the ED-psychopathology. Similar to our study, these findings’ clinical implications rely on understanding this psychological profile, underlying eating symptoms, in order to prevent relapses.

Monteleone and colleagues applied for the first time a network approach to bariatric surgery candidates to explore the interplay between eating symptoms, personality traits, and anxiety. In line with our findings, their study stressed the importance of incorporating psychological variables into the pre-surgery assessment, as well as considering them as potential targets for psychotherapeutic interventions before and after surgery (31) Instead, differently from our study, their research includes the administration of a specific instrument, the Temperament and Character Inventory-Revised (TCI-R) (40) that comprehensively assesses the patient’s personality profile, allowing a detailed overview of the personological traits of this clinical population. They also combined the network analysis approach with prognostic data. A deeper personological assessment and a longitudinal perspective could therefore be future perspectives for our study. Calugi & Dalle Grave used a network analysis to assess the interplay between psychosocial variables in a large sample of patients with obesity, stressing how this innovative approach allows an in-depth exploration of their complex clinical profile (38). In contrast to our study, their sample does not include bariatric patients and they have only evaluated psychosocial factors associated with obesity, not including personality traits. Their network analysis, however, considered also the internalized weight stigma in patients seeking treatment for obesity, which emerged as one of the most relevant variable in their network, together with interpersonal sensitivity, and shape-weight concern. A growing body of research (32–38) applied network analysis to EDs, even though not specifically on bariatric surgery candidates, their implications are consistent with ours in stressing the interplay between eating behaviors and psychopathological traits and the need to implement a network approach. Our results confirm the need for a multidisciplinary approach to bariatric surgery, implementing psychiatric and psychological interventions in order to discover and treat pathological eating behaviors and psychopathological traits such as emotion dysregulation, impulsivity, anxiety, and depression, that can be either the cause of obesity or of weight-regain (41, 42).

The present network analysis suggests that emotion dysregulation links the relationship between dysfunctional eating patterns and symptoms of anxiety and depression. In conclusion, this study highlights the importance of a comprehensive psychological assessment for patients seeking bariatric surgery as it identifies the presence of multiple pathological eating behaviors and psychopathological traits. In this regard, the present study stresses the utility of a network approach to understand the psychological background of bariatric candidates and develop tailored interventions that address specific nodes in the network, potentially improving the long-term success of bariatric surgery and reducing the risk of relapse into pathological eating behaviors.

### 4.1 Clinical implications

Our findings have quickly translatable clinical implications, because interventions on emotion regulation can act as therapeutic targets to break the vicious cycle between dysfunctional eating patterns, emotion dysregulation, and anxious-depressive symptoms in line with previous research (43). This also suggests that interventions that target impulsivity and emotion regulation, such as cognitive-behavioral therapy (44) or mindfulness-based interventions (45), may be beneficial in treating pathological eating behaviors in patients seeking bariatric surgery (e.g., mindfulness for emotional eating) (46).

### 4.2 Limitations

The cross-sectional design and reliance on self-report questionnaires may represent limitations of the study’s ability to infer causality or temporal dynamics and the monocentric nature of the study may limit its generalizability to other populations. Further research is needed to investigate the associations between eating styles and psychopathological traits, particularly in relation to the risk of excessive weight gain or dysfunctional eating following bariatric surgery and to test the replicability of these findings in other ethnic groups, as well as address potential sources of inequality. It is crucial to assess whether emotion regulation difficulties persist or re-emerge after surgery and their impact on pathological eating styles and weight outcomes in the long term. In particular, longitudinal randomized control trials are needed to evaluate whether treatments targeting emotion regulation difficulties and/or dysfunctional eating styles may improve the outcomes of bariatric surgery, as well as patients’ psychopathological traits.

## Data availability statement

The raw data supporting the conclusions of this article will be made available by the authors, without undue reservation.

## Ethics statement

The studies involving humans were approved by Ethics Committee of Fatebenefratelli Sacco Hospital (“psycBAR36”‘, 2020). The studies were conducted in accordance with the local legislation and institutional requirements. The participants provided their written informed consent to participate in this study.

## Author contributions

LS: Data curation, Visualization, Writing – original draft, Writing – review & editing. AB: Conceptualization, Investigation, Writing – original draft, Writing – review & editing. PL: Conceptualization, Investigation, Methodology, Supervision, Writing – review & editing. MS: Conceptualization, Investigation, Methodology, Writing – review & editing. MZ: Conceptualization, Funding acquisition, Methodology, Writing – review & editing. BD: Conceptualization, Funding acquisition, Methodology, Writing – review & editing. GR: Data curation, Formal analysis, Software, Supervision, Visualization, Writing – review & editing.
