# Corrigendum: Emotion dysregulation links pathological eating styles and psychopathological traits in bariatric surgery candidates

**DOI:** 10.3389/fpsyt.2024.1495351

**Published:** 2024-09-30

**Authors:** Arianna Belloli, Luigi F. Saccaro, Paola Landi, Milena Spera, Marco Antonio Zappa, Bernardo Dell’Osso, Grazia Rutigliano

**Affiliations:** ^1^ Department of Psychiatry, Azienda Socio Sanitaria Territoriale (ASST) Fatebenefratelli-Sacco, Milan, Italy; ^2^ Department of Psychology, Sigmund Freud University, Milan, Italy; ^3^ Department of Psychiatry, Faculty of Medicine, University of Geneva, Geneva, Switzerland; ^4^ Department of Psychiatry, Geneva University Hospital, Geneva, Switzerland; ^5^ Department of General Surgery, Azienda Socio Sanitaria Territoriale (ASST) Fatebenefratelli-Sacco, Milan, Italy; ^6^ Institute of Clinical Sciences, Faculty of Medicine, Imperial College London, London, United Kingdom

**Keywords:** obesity, bariatric surgery, eating style, emotion regulation, network analysis

In the published article, there was an error in the legends for **Figures 1** and **2** as published. The last part of both figure legends originally read: “The color of the edge indicates the size of the association (blue for positive associations; red for negative associations).”

**Figure 1:**
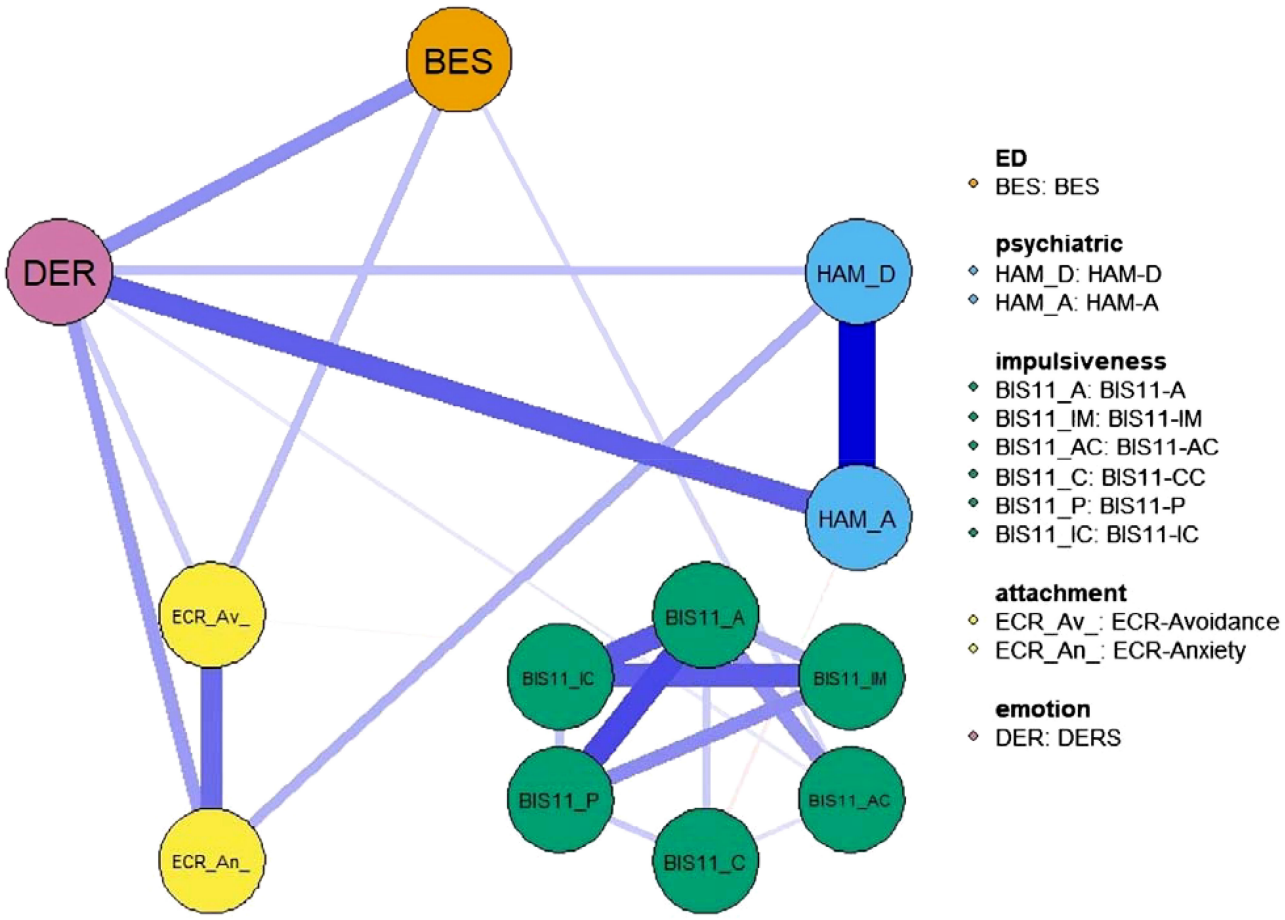
Network structure. The variables include eating disorder-specific scales (BES), psychiatric scales (HAM-D and HAM-A) and psychological and personality scales (BIS-11, ECR and DER). Item groups are differentiated by color. Edge colors represent the direction of associations (blue for positive, red for negative), and edge widths indicate the strength of these associations.

**Figure 2:**
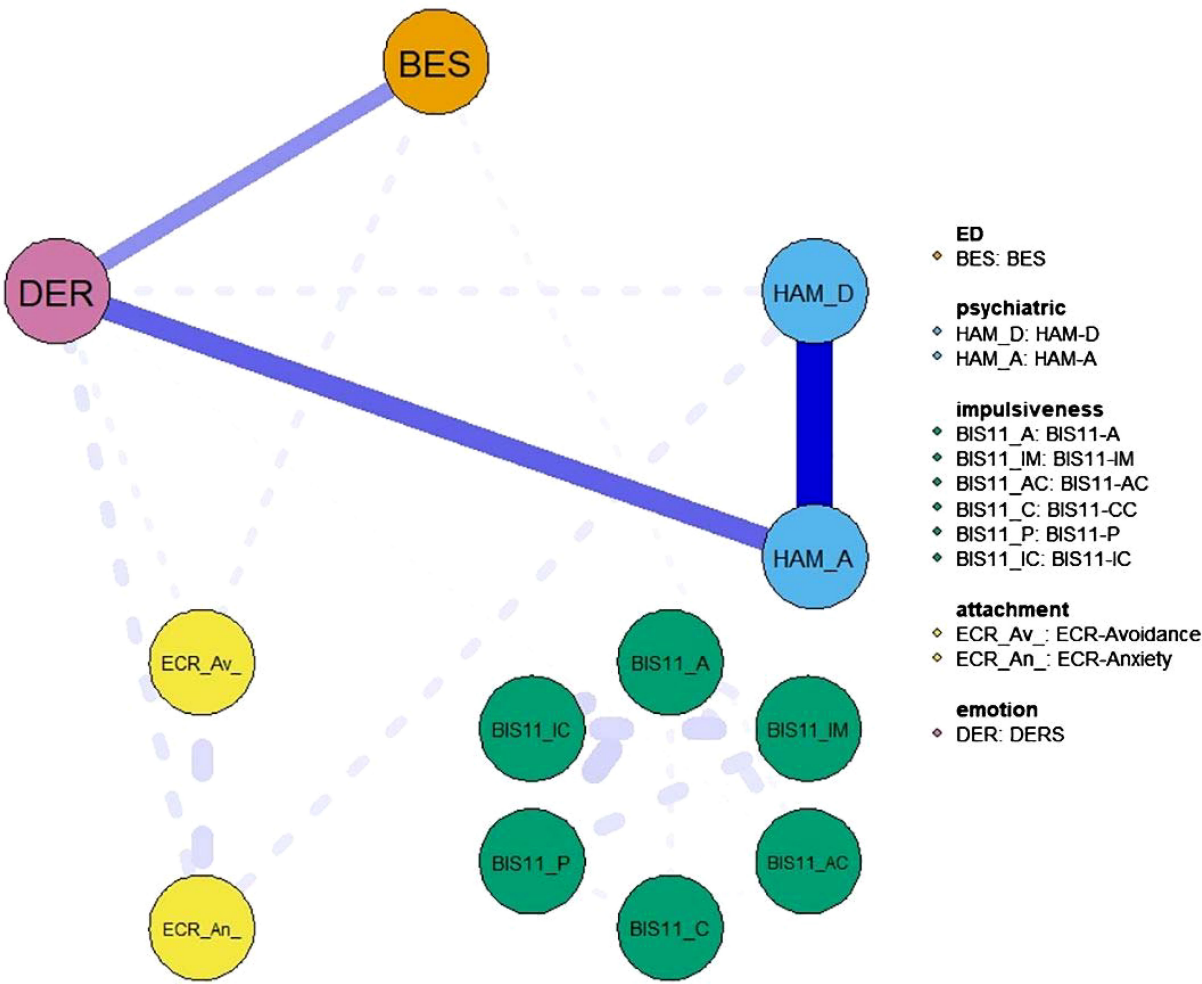
Network showing the shortest path between the BES and Hamilton depression and anxiety scales. The variables include eating disorder-specific scales (BES), psychiatric scales (HAM-D and HAM-A) and psychological and personality scales (BIS-11, ECR and DER). Item groups are differentiated by color. Edge colors represent the direction of associations (blue for positive, red for negative), and edge widths indicate the strength of these associations.

The corrected legends appear below.

FIGURE 1 Network structure. The variables include eating disorder-specific scales (BES), psychiatric scales (HAM-D and HAM-A) and psychological and personality scales (BIS-11, ECR and DER). Item groups are differentiated by color. Edge colors represent the direction of associations (blue for positive, red for negative), and edge widths indicate the strength of these associations.

FIGURE 2 Network showing the shortest path between the BES and Hamilton depression and anxiety scales. The variables include eating disorder-specific scales (BES), psychiatric scales (HAM-D and HAM-A) and psychological and personality scales (BIS-11, ECR and DER). Item groups are differentiated by color. Edge colors represent the direction of associations (blue for positive, red for negative), and edge widths indicate the strength of these associations.

The authors apologize for this error and state that this does not change the scientific conclusions of the article in any way. The original article has been updated.

