# Supplementary material for: Emotion dysregulation links pathological eating styles and psychopathological traits in bariatric surgery candidates
Source: Front Psychiatry. 2024 Mar 28;15:1369720. doi: 10.3389/fpsyt.2024.1369720 (PMC11006956; doi:10.3389/fpsyt.2024.1369720)
Supplement: Supplementary file 1 [file DataSheet_1.docx]

Supplementary Material

**1.** **Details of clinical scores**

*1.1.* *Difficulties in Emotion Regulation Scale* (DERS) (1)

DERS is a 36-item self-report measure that assesses difficulties in emotion regulation across multiple domains, with higher scores indicating greater difficulties in emotion regulation. This tool allows to obtain measurements regarding the presence of potential difficulties in the following dimensions: (a) awareness and understanding of emotions, (b) acceptance of emotions, (c) ability to control impulsive behaviors and to behave in accordance with their goals and (d) ability to use flexible emotional regulation strategies appropriate to the context and to the situational demands. It Contains six subscales: (i) NON-ACCEPTANCE (non-acceptance of emotional responses) is formed by items that reflect the tendency to experience negative secondary emotions in response to one's negative emotions, or to have reactions to non-acceptance of their own discomfort, (ii) GOALS (difficulty in adopting behaviors goal-oriented) includes items reflecting difficulties in concentrating in performing a task when negative emotions are experienced, (iii) IMPULSE (i.e. difficulty in impulse control) notes the difficulty in maintaining control of one's behavior when experiencing negative emotions, (iv) AWARENESS (lack of emotional awareness) contains items that underline the tendency to pay attention to emotions and the relative capacity to recognize them (for this reason the answers provided must be reversed in the calculation phase of the score), (v) STRATEGIES (limited access to emotional regulation strategies) reflect the belief that it is particularly difficult to effectively regulate emotions once they have manifested, (vi) CLARITY (lack of emotional clarity) includes items which reflect the degree to which people can distinctly understand which emotion they are experimenting.

The 36 DERS items are scored from 1 to 5, where 1 is almost never (0-10%), 2 is sometimes (11-35%), 3 is about half the time (36-65%), 4 is most of the time (66-90%), and 5 is almost always (91-100%). Of the 36 items, 11 are reverse scored.

The DERS has been found to have high internal consistency, good test-retest reliability, and adequate construct and predictive validity.

Scores are presented as total scores and as scores for each of the 6 subscales. Higher scores indicate greater problems with emotion regulation. Total scores range from 36 to 180. The clinical range of the DERS total score varies from an average of about 80 to 127 and difficulties in emotional regulation can be scored as Mild (if < 90), Moderate (91-105), or Severe (>105).

*1.2.* *Hamilton Depression Rating Scale* (HAM-D) (2)

The questionnaire is designed for adults and is used to rate the severity of their depression by probing mood, feelings of guilt, suicide ideation, insomnia, agitation or retardation, anxiety, weight loss, and somatic symptoms. Each item on the questionnaire is scored on a 3 or 5 point scale. Depression is detected if the total score is >7 with the following classifications: Mild (subthreshold): 8–13, Moderate (mild): 14–18, Severe (moderate): 19–22, Very severe (severe): >23.

*1.3.* *Hamilton Anxiety Rating Scale* (HAM-A) (3)

The questionnaire rates the severity of a patient's anxiety. It consists of 14 items designed to assess the severity of a patient's anxiety. Each of the 14 items contains a number of symptoms, and each group of symptoms is rated on a Likert scale 1-4, with 4 being the most severe. A score of 17 or less indicates mild anxiety severity, scores from 25 to 30 indicates moderate to severe anxiety severity.

*1.4. Barratt Impulsiveness Scale* (BIS-11) (4)

This clinical scale measures the patient's impulsiveness levels. It is made up of 30 items, which are scored on a Likert scale 1-4 (Rarely/Never = 1, Occasionally = 2, Often = 3, Almost Always/Always = 4). Scores range from 30 to 120. There are no standardized cut-off scores, but a score between 70 and 75 could indicate pathological traits of subclinical impulsivity, a score > 75 could indicate an impulse control disorder and finally a range > 70 is not clinically relevant. The 30 items refer to six first-order factors that include: Attention (BIS-11, A), Motor impulsiveness (BIS-11, Im), Self-control (BIS-11, Ac), Cognitive complexity (BIS-11, Cc), Perseverance (BIS-11, P) and Cognitive Instability (BIS-11, IC).

*1.5. Binge Eating Scale* (BES) (5)

This self-report 16-item questionnaire is aimed at assessing episodes of food dyscontrol (binge eating episodes) and the related emotional arousal. The score range is from 0 to 46. A score < 17 indicates the absence of binge, a score between 18-26 means a moderate binge and a score > 27 corresponds to the presence of binge eating. This clinical scale evaluates behavioral, cognitive and emotional variables linked to food dyscontrol, with a focus on guilt and shame.

*1.6. Experiences in Close Relationships Questionnaire - Revised* (ECR-R) (6)

ECR-R is a 36-item self-report measure of adult attachment style, whose items are scored on a Likert scale 1-7 (Strongly disagree = 1-3, Neither disagree, nor agree = 4-6, Strongly agree = 7). It is made up of two subscales of attachment styles: Anxiety and Avoidance. The first 18 items belong to the anxiety-related subscale, while items 19–36 to the avoidance-related one. To obtain a score for attachment-related anxiety, you have to average items from 1 to 18 and do the same for attachment-related avoidance with items from 19 to 36, taking into account the reversed items. The cut-offs of this scale depend on the age of the subject:

- 20 years old: Avoidance (2.90); Anxiety (3.67)
- 30 years old: Avoidance (2.97); Anxiety (3.56)
- 40 years old: Avoidance (3.04); Anxiety (3.45)
- 50 years old: Avoidance (3.11); Anxiety (3.34)
- 60 years old: Avoidance (3.18); Anxiety (3.23)

*1.7.* *Body mass index*

Measures of height and weight were collected by a bariatric dietician, and body mass index (BMI) was calculated as weight [kg]/height^2^ [m^2^].

*1.8.* *Eating behavioral styles*

Along with clinical scores, patients were thoroughly monitored during a multi-disciplinary consultation in order to determine surgery eligibility. At first, in brief, smoking, drinking, and eating habits were examined through a general food diary (breakfast, snack, lunch, dinner). Then, through a detailed clinical interview with specific questions for each eating style (**Supplementary Table 1**), a psychiatrist (PL) and psychotherapist (MS) clinically assessed and classified the eating behavior styles of each subject based on their symptoms and clinical history. The eating behavioral styles are defined as follows. Emotional eating commonly refers to abnormal eating in reaction to (predominantly) negative emotions (e.g. stress, anger, fear, boredom, sadness and loneliness). It is a maladaptive way of coping and is thought to represent more extensive problems with emotional regulation, which may be themselves features and risk factors for severe psychiatric disorders. Emotional eating is one of the most frequent maladaptive eating behaviors in bariatric patients and one of the major causes of weight regain after surgery, since emotional regulation problems usually remain unaltered over time and associate with binge eating (7). Binge eating is an uncontrolled intake of food, resulting from a sudden unavoidable need, with the impossibility of stopping, often with ingestion of both sweet and savory foods, up to a sensation of extreme gastric fullness. It is characterized by the presence of high levels of impulsiveness. Qualitative (or external) eating refers to abnormal eating in response to qualitative aspects of the food, e.g. the smell, taste, or appearance of food. Gorging refers to the ingestion of a large amount of food, usually coinciding with the main meals, while snacking defines the ingestion of quantities of food mainly between meals.

**2.** **SICOb criteria**

According to the SICOb (the Italian Society of Surgery for Obesity and Metabolic Diseases), bariatric surgery is indicated in patients with:

• BMI> 40 kg / m2, in the absence of any other comorbidities;

• BMI> 35 kg / m2, in the presence of comorbidities among those classically considered rates as associated with obesity, including type 2 diabetes mellitus (T2DM) resistant to medical treatment.

**SUPPLEMENTARY FIGURES**


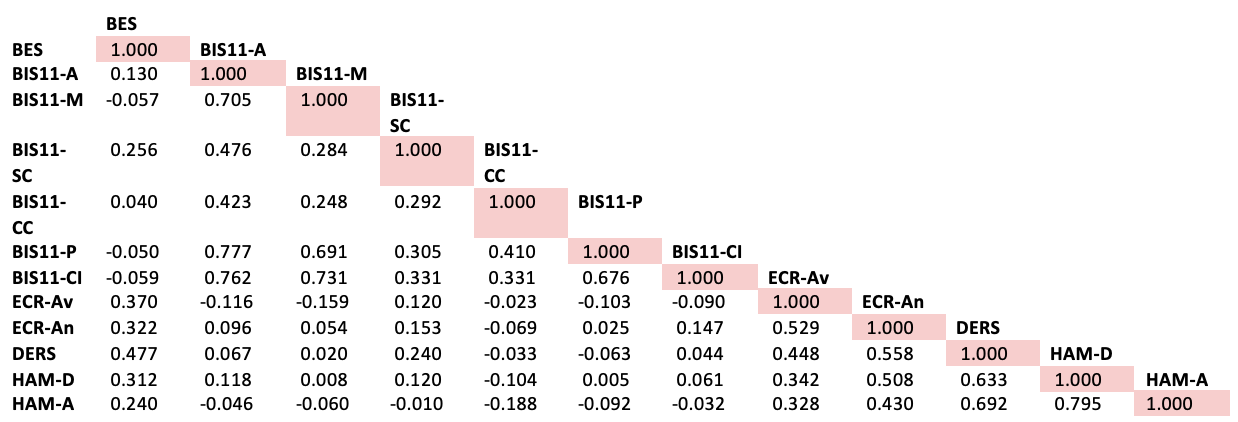


**Supplementary Figure 1. Correlation matrix among all the clinical variables.**

**
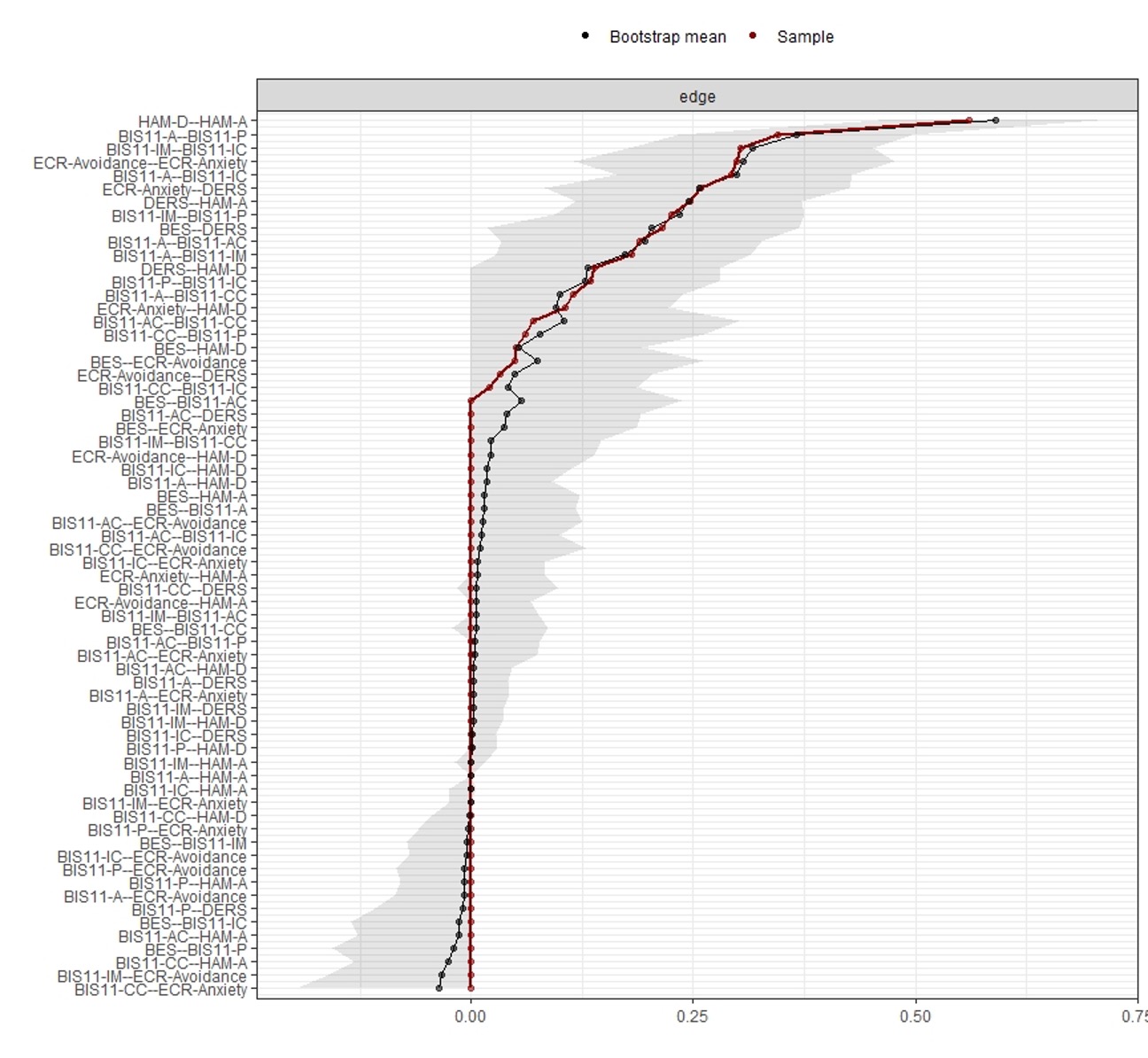
**

**Supplementary Figure 2. Accuracy of the edge-weights.** Bootstrapped confidence intervals of estimated edge-weights for the estimated network of ED-specific scales (BES), psychiatric scales (HAM-D and HAM-A) and psychological and personality scales (BIS-11, ECR and DERS) in our sample of patients candidate to bariatric surgery. The horizontal area within the plot represents the 95% quartile range of the parameter values across 2500 bootstraps. The red dots indicate the sample values for the analyzed data, while the black dots indicate the bootstrap mean values. The sample values lie within the bootstrapped confidence intervals and the bootstrap mean values are generally well-aligned with the sample values, thus indicating accurate estimations. Of note, the bootstrapped confidence intervals are relatively wide, thus some caution is recommended especially when interpreting the presence and strength of weaker edges.

**
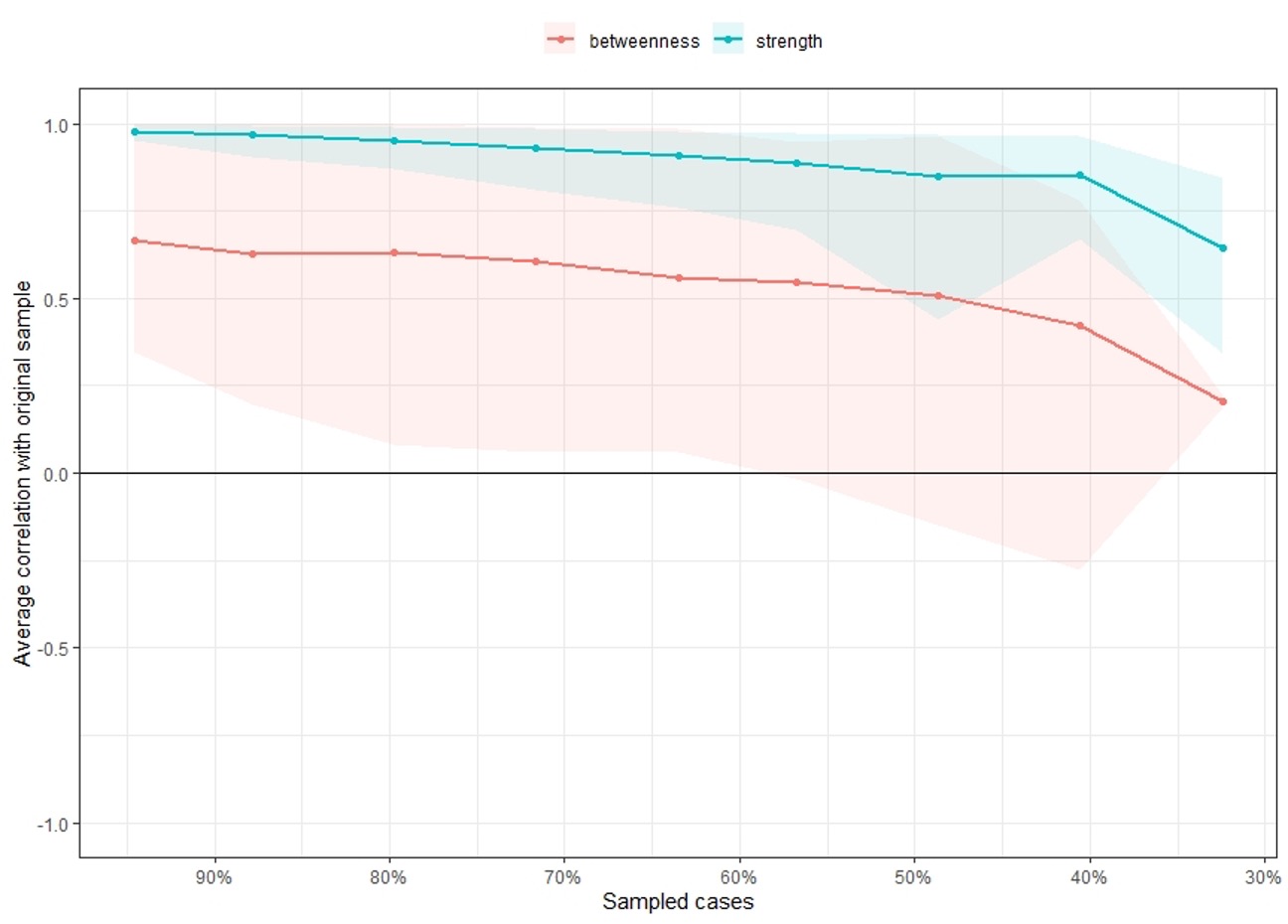
**

**Supplementary Figure 3.** Average correlations between centrality indices (strength and betweenness) of networks sampled when dropping different proportions of the data, based on 2500 iterations. Lines indicate the means and areas indicate the 95% confidence intervals. Central stability coefficient (maximum drop proportions to retain correlation of 0.7 in at least 95% of the sample) was 0.365 for strength and 0 for betweenness (networks with reliable centrality should have a stability coefficient greater or equal to 0.25, ideally higher than 0.5 for centrality estimates).

**Networks showing the shortest paths between the BES and each of the following:**

*1. BIS-11*


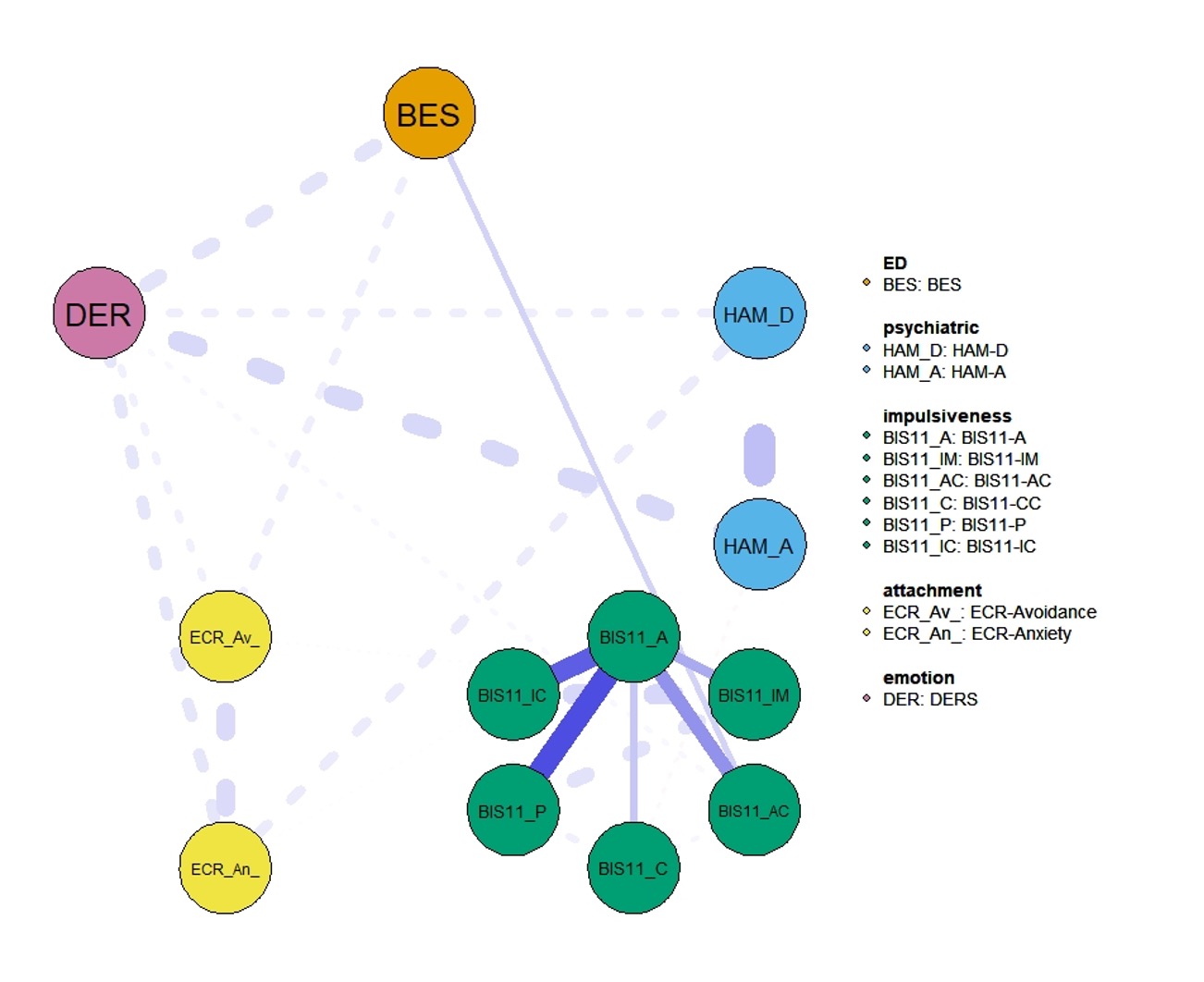


**Supplementary Figure 4. shortest paths between the BES and BIS-11**

*2. Attachment*


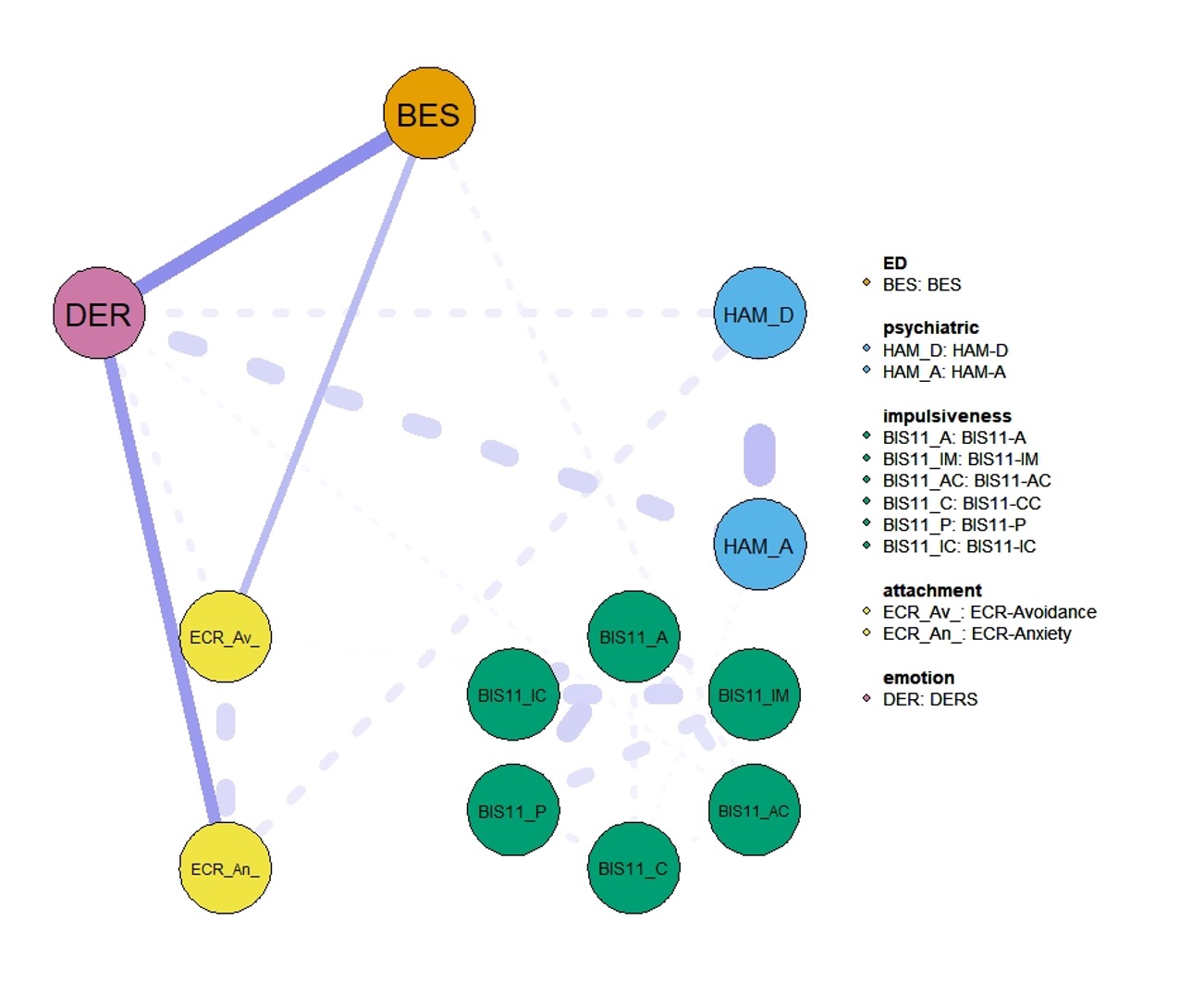


**Supplementary Figure 5. shortest paths between the BES and ECR**

*3. DERS*


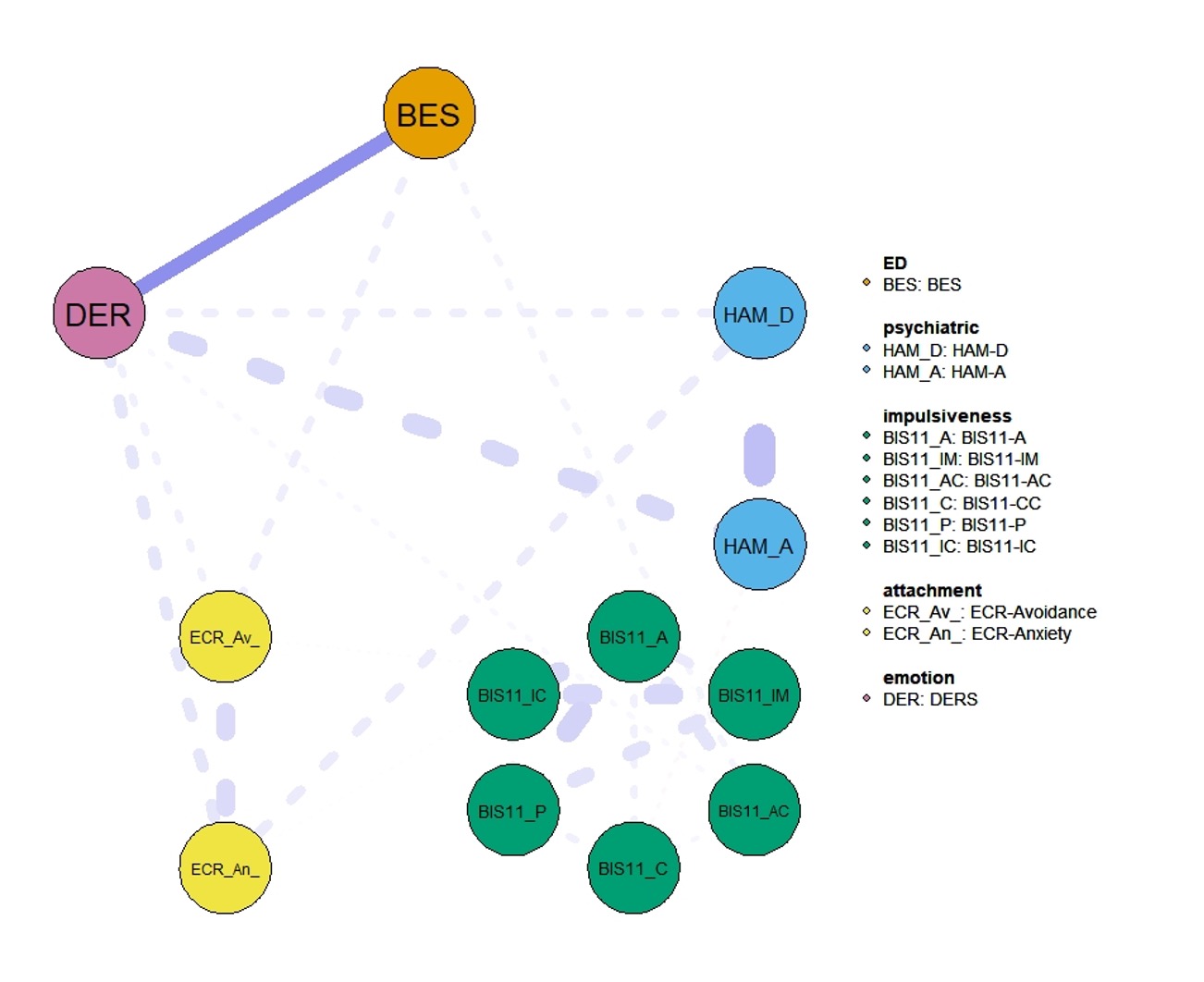


**Supplementary Figure 6. shortest paths between the BES and DERS**

**SUPPLEMENTARY TABLES**

**Supplementary Table 1. Assessment of eating behavioral styles.**

| **EATING STYLE** | **EXAMPLES OF CLINICAL QUESTIONS** |
| --- | --- |
| Emotional | Does it occur often that a sudden, relentless need of eating arises, satisfied by the ingestion of both sweet and savory foods, until a sensation of extreme stomach fullness? |
| Binge | Do you consume large quantities of food in a short period of time with the feeling of loss of control, i.e. of not being able to retain yourself? |
| Qualitative | -Do you find more gratification from a plate overflowing with boiled rice or from a small but tasty food?  -Would you say that, for you, food quality (i.e. its taste, appearance, smell…) is remarkably more satisfying than its quantity and it leads you to inappropriate or exaggerate consumption of food based on its quality? |
| Quantitative | -Do you find more gratification from a plate overflowing with boiled rice or from a small but tasty food?  - Would you say that, for you, quantity of food is remarkably more satisfying than its qualitative characteristics (i.e. its taste, appearance, smell…) quantity and it leads you to inappropriate or exaggerate consumption of food based on its quantity? |
| Gorge | Based on quantity only, would you say that you eat until you feel your stomach tensed and extremely full? |
| Snack | Do you frequently and regularly eat snacks, intended as small quantities of food, between main daily meals? |

**Supplementary Tables 2 a-f. Differences in clinical measures among eating styles.**

The following supplementary tables describe results on differences in clinical measures among eating styles.

**Supplementary Table** **2a**. Differences in eating-disorder-specific factors (BES), psychological and personality factors (BIS-11, ECR, DERS) and psychiatric factors (HAM-D, HAM-A) by quantitative Eating Style.

| Eating Style, Quantity | Yes | | No | |  |
| --- | --- | --- | --- | --- | --- |
|  | median | 25%-75% percentile | median | 25%-75% percentile | p-value |
| **BES** | 13.00 | 8.00-20.00 | 10.00 | 4.00-18.50 | 0.191 |
| **BIS-11, A** | 14.00 | 10.00-16.00 | 12.00 | 7.00-15.00 | 0.122 |
| **BIS-11, Im** | 19.00 | 12.00-23.50 | 19.00 | 12.00-24.00 | 0.916 |
| **BIS-11, Ac** | 14.00 | 11.00-15.50 | 12.00 | 10.00-14.00 | 0.173 |
| **BIS-11, Cc** | 13.00 | 12.00-15.00 | 13.00 | 11.00-15.00 | 0.620 |
| **BIS-11, P** | 11.00 | 7.00-13.00 | 11.00 | 6.00-13.00 | 0.679 |
| **BIS-11, Ic** | 8.00 | 6.00-10.00 | 7.00 | 6.00-10.00 | 0.936 |
| **BIS-11, Total** | 79.00 | 58.00-89.50 | 82.00 | 51.00-87.00 | 0.458 |
| **ECR-Av-mean** | 2.93 | 2.22-3.56 | 3.09 | 2.30-3.81 | 0.355 |
| **ECR-An-mean** | 5.58 | 4.16-7.08 | 5.28 | 4.06-7.63 | 0.831 |
| **ECR-Av** | 53.00 | 39.00-64.00 | 54.50 | 38.75-68.75 | 0.615 |
| **ECR-An** | 53.00 | 39.50-65.50 | 46.00 | 31.50-72.50 | 0.622 |
| **DERS** | 74.00 | 59.00-93.75 | 80.50 | 66.75-107.25 | 0.222 |
| **HAM-D** | 9.00 | 5.00-14.00 | 14.00 | 4.00-18.00 | 0.138 |
| **HAM-A** | 9.00 | 3.00-12.00 | 12.00 | 7.25-19.00 | ***0.014*** |

**Supplementary table** **2b**. Differences in eating-disorder-specific factors (BES), psychological and personality factors (BIS-11, ECR, DERS) and psychiatric factors (HAM-D, HAM-A) by qualitative Eating Style.

| Eating Style,  Quality | Yes | | No | |  |
| --- | --- | --- | --- | --- | --- |
|  | median | 25%-75% percentile | median | 25%-75% percentile | p-value |
| **BES** | 13.00 | 8.00-15.00 | 10.00 | 5.00-17.00 | 0.112 |
| **BIS-11, A** | 11.00 | 8.00-15.00 | 15.00 | 11.00-16.50 | ***0.006*** |
| **BIS-11, Im** | 16.00 | 11.00-21.00 | 22.00 | 14.50-25.50 | ***0.003*** |
| **BIS-11, Ac** | 13.00 | 10.00-16.00 | 14.00 | 11.00-15.00 | 0.361 |
| **BIS-11, Cc** | 13.00 | 11.00-15.00 | 14.00 | 12.00-15.00 | 0.545 |
| **BIS-11, P** | 9.00 | 7.00-12.00 | 12.00 | 8.50-14.00 | ***0.010*** |
| **BIS-11, Ic** | 7.00 | 5.00-10.00 | 10.00 | 7.00-10.00 | ***0.026*** |
| **BIS-11, Total** | 71.00 | 54.00-87.00 | 86.00 | 72.00-91.50 | ***0.008*** |
| **ECR-Av-mean** | 3.00 | 2.26-3.59 | 2.94 | 2.11-3.64 | 0.766 |
| **ECR-An-mean** | 5.58 | 4.00-7.69 | 5.64 | 4.71-6.03 | 0.907 |
| **ECR-Av** | 54.00 | 40.50-64.50 | 52.50 | 36.75-65.00 | 0.492 |
| **ECR-An** | 53.00 | 38.00-73.00 | 51.00 | 42.75-57.00 | 0.628 |
| **DERS** | 77.00 | 63.00-98.25 | 70.50 | 59.00-93.75 | 0.317 |
| **HAM-D** | 9.00 | 4.25-17.00 | 9.00 | 5.00-14.00 | 0.591 |
| **HAM-A** | 11.00 | 5.00-15.00 | 8.00 | 3.00-13.00 | 0.195 |

**Supplementary table** **2c**. Differences in eating-disorder-specific factors (BES), psychological and personality factors (BIS-11, ECR, DERS) and psychiatric factors (HAM-D, HAM-A) by Snacking Eating Style.

| Eating Style,  Snack | Yes | | No | |  |
| --- | --- | --- | --- | --- | --- |
|  | median | 25%-75% percentile | median | 25%-75% percentile | p-value |
| **BES** | 14.00 | 8.00-21.00 | 12.00 | 4.50-16.00 | 0.059 |
| **BIS-11, A** | 14.00 | 9.25-16.00 | 13.00 | 9.25-15.75 | 0.634 |
| **BIS-11, Im** | 18.50 | 12.00-23.00 | 20.00 | 11.25-24.00 | 0.861 |
| **BIS-11, Ac** | 13.50 | 11.00-15.75 | 14.00 | 10.00-15.00 | 0.708 |
| **BIS-11, Cc** | 13.50 | 12.00-15.00 | 13.00 | 10.25-14.75 | 0.138 |
| **BIS-11, P** | 10.50 | 7.00-13.00 | 11.00 | 7.00-13.00 | 0.499 |
| **BIS-11, Ic** | 7.50 | 6.00-10.00 | 8.00 | 6.00-10.00 | 0.773 |
| **BIS-11, Total** | 78.50 | 58.50-89.75 | 81.50 | 54.75-87.75 | 0.802 |
| **ECR-Av-mean** | 2.83 | 2.11-3.50 | 3.09 | 2.32-3.87 | 0.099 |
| **ECR-An-mean** | 5.47 | 4.06-6.11 | 5.68 | 4.66-7.92 | 0.231 |
| **ECR-Av** | 45.00 | 36.50-61.00 | 55.50 | 41.75-68.25 | 0.076 |
| **ECR-An** | 49.00 | 37.50-58.00 | 53.50 | 42.75-75.25 | 0.222 |
| **DERS** | 76.00 | 60.00-99.00 | 77.00 | 63.50-94.00 | 0.992 |
| **HAM-D** | 8.50 | 4.00-17.00 | 10.00 | 6.00-16.00 | 0.431 |
| **HAM-A** | 9.00 | 4.00-14.00 | 9.00 | 3.50-13.50 | 0.985 |

**Supplementary table** **2d**. Differences in eating-disorder-specific factors (BES), psychological and personality factors (BIS-11, ECR, DERS) and psychiatric factors (HAM-D, HAM-A) by Gorging Eating Style.

| Eating Style,  Gorge | Yes | | No | |  |
| --- | --- | --- | --- | --- | --- |
|  | median | 25%-75% percentile | median | 25%-75% percentile | p-value |
| **BES** | 15.00 | 7.75-21.00 | 11.00 | 6.00-16.25 | 0.190 |
| **BIS-11, A** | 15.00 | 11.00-16.00 | 11.00 | 8.00-15.00 | ***0.012*** |
| **BIS-11, Im** | 21.50 | 16.00-24.75 | 16.00 | 12.00-21.75 | ***0.029*** |
| **BIS-11, Ac** | 14.00 | 12.00-17.00 | 11.50 | 10.00-14.00 | ***0.001*** |
| **BIS-11, Cc** | 13.00 | 12.00-15.00 | 13.00 | 11.00-15.00 | 0.632 |
| **BIS-11, P** | 12.00 | 8.00-14.00 | 9.00 | 7.00-12.00 | ***0.031*** |
| **BIS-11, Ic** | 9.00 | 6.25-10.00 | 7.00 | 5.00-10.00 | 0.119 |
| **BIS-11, Total** | 85.50 | 71.00-91.75 | 68.00 | 54.00-86.75 | ***0.011*** |
| **ECR-Av-mean** | 2.97 | 2.32-3.53 | 3.00 | 2.00-3.70 | 0.721 |
| **ECR-An-mean** | 5.58 | 4.22-7.07 | 5.60 | 3.84-7.42 | 0.925 |
| **ECR-Av** | 53.50 | 41.75-63.50 | 53.00 | 35.50-66.50 | 0.586 |
| **ECR-An** | 51.00 | 39.75-67.00 | 52.00 | 33.50-70.50 | 0.976 |
| **DERS** | 74.50 | 63.25-95.75 | 76.50 | 60.25-97.00 | 0.793 |
| **HAM-D** | 9.00 | 5.00-16.00 | 9.50 | 4.00-16.25 | 0.682 |
| **HAM-A** | 9.00 | 3.00-13.50 | 10.00 | 5.00-14.00 | 0.356 |

**Supplementary table** **2e**. Differences in eating-disorder-specific factors (BES), psychological and personality factors (BIS-11, ECR, DERS) and psychiatric factors (HAM-D, HAM-A) by Binge Eating Style.

| Eating Style,  Binge | Yes | | No | |  |
| --- | --- | --- | --- | --- | --- |
|  | median | 25%-75% percentile | median | 25%-75% percentile | p-value |
| **BES** | 21.00 | 14.75-26.00 | 11.00 | 5.25-16.75 | **<0.001** |
| **BIS-11, A** | 15.00 | 10.00-16.00 | 13.00 | 9.00-16.00 | 0.409 |
| **BIS-11, Im** | 19.00 | 12.00-22.00 | 19.00 | 12.00-24.00 | 0.863 |
| **BIS-11, Ac** | 15.00 | 12.00-17.00 | 13.00 | 11.00-15.00 | 0.086 |
| **BIS-11, Cc** | 13.00 | 11.00-14.00 | 13.00 | 11.50-15.00 | 0.224 |
| **BIS-11, P** | 9.00 | 7.00-12.00 | 11.00 | 7.00-13.00 | 0.540 |
| **BIS-11, Ic** | 10.00 | 5.00-11.00 | 8.00 | 6.00-10.00 | 0.450 |
| **BIS-11, Total** | 83.00 | 58.00-92.00 | 80.00 | 57.00-88.50 | 0.791 |
| **ECR-Av-mean** | 3.50 | 3.22-3.83 | 2.91 | 2.22-3.56 | ***0.038*** |
| **ECR-An-mean** | 6.00 | 5.68-7.89 | 5.16 | 4.00-7.00 | ***0.019*** |
| **ECR-Av** | 63.00 | 58.00-69.00 | 51.50 | 38.00-64.00 | ***0.026*** |
| **ECR-An** | 57.00 | 54.00-75.00 | 47.00 | 37.25-64.75 | ***0.017*** |
| **DERS** | 96.00 | 71.75-117.00 | 74.50 | 59.75-90.25 | ***0.038*** |
| **HAM-D** | 14.50 | 9.00-18.75 | 9.00 | 4.00-16.00 | ***0.012*** |
| **HAM-A** | 12.00 | 9.25-27.75 | 9.00 | 3.00-13.00 | ***0.012*** |

**Supplementary table** **2f**. Differences in eating-disorder-specific factors (BES), psychological and personality factors (BIS-11, ECR, DERS) and psychiatric factors (HAM-D, HAM-A) by Emotional Eating Style.

| Eating Style,  Emotional Eating | Yes | | No | |  |
| --- | --- | --- | --- | --- | --- |
|  | median | 25%-75% percentile | median | 25%-75% percentile | p-value |
| **BES** | 13.00 | 8.00-21.00 | 10.00 | 4.00-16.00 | ***0.030*** |
| **BIS-11, A** | 14.00 | 10.00-16.00 | 13.00 | 8.25-16.00 | 0.564 |
| **BIS-11, Im** | 19.00 | 12.00-23.00 | 17.50 | 12.00-24.00 | 0.894 |
| **BIS-11, Ac** | 14.00 | 11.00-16.00 | 12.50 | 11.00-14.75 | 0.456 |
| **BIS-11, Cc** | 13.00 | 12.00-14.75 | 13.00 | 10.25-15.00 | 0.886 |
| **BIS-11, P** | 11.00 | 7.00-12.00 | 10.00 | 7.00-14.00 | 0.703 |
| **BIS-11, Ic** | 8.00 | 6.00-10.00 | 7.00 | 5.25-10.00 | 0.745 |
| **BIS-11, Total** | 81.50 | 58.25-88.75 | 76.00 | 53.25-91.00 | 0.831 |
| **ECR-Av-mean** | 3.17 | 2.24-3.83 | 2.72 | 2.10-3.16 | 0.051 |
| **ECR-An-mean** | 5.70 | 4.74-7.26 | 4.69 | 3.21-5.95 | ***0.009*** |
| **ECR-Av** | 56.00 | 40.00-68.00 | 49.00 | 37.25-56.75 | 0.102 |
| **ECR-An** | 54.00 | 43.00-69.00 | 44.50 | 30.50-56.50 | ***0.028*** |
| **DERS** | 77.50 | 64.25-100.50 | 67.50 | 56.00-87.75 | 0.056 |
| **HAM-D** | 12.00 | 6.00-17.25 | 5.00 | 3.00-9.75 | ***0.001*** |
| **HAM-A** | 11.00 | 5.00-17.00 | 5.00 | 3.00-11.00 | ***0.001*** |

**Supplementary table** **3**. Details on missing values per variable. N: number.

| **Variable** | **Missing** | | **Valid N** |
| --- | --- | --- | --- |
|  | **N** | **Percent** |  |
| Difficulties Emotional Regulation Scale | 32 | 29.1% | 78 |
| Experience Close Relationship_Anxiety | 31 | 28.2% | 79 |
| Experience Close Relationship_Avoidance | 31 | 28.2% | 79 |
| Barratt Impulsiveness Scale_Total | 26 | 23.6% | 84 |
| Barratt Impulsiveness Scale_MotorImpulsiveness | 26 | 23.6% | 84 |
| Barratt Impulsiveness Scale_CognitiveImpulsiveness | 26 | 23.6% | 84 |
| BarrattImpulsivenessScale_Perseverance | 26 | 23.6% | 84 |
| BarrattImpulsivenessScale_CognitiveComplexity | 26 | 23.6% | 84 |
| BarrattImpulsivenessScale_SelfControl | 26 | 23.6% | 84 |
| BarrattImpulsivenessScale_Attention | 26 | 23.6% | 84 |
| BingeEatingScale | 16 | 14.5% | 94 |
| Hamilton_Depression | 15 | 13.6% | 95 |
| Hamilton_Anxiety | 14 | 12.7% | 96 |

**Supplementary table** **4**. Results of logistic regressions. Confidence Interval (CI), Difficulties in Emotion Regulation Scale (DERS), Experiences in Close Relationships Questionnaire - Revised-Avoidance/Anxiety (ECR-Av/An), Hamilton Depression Scale (HAM-D), and Hamilton Anxiety Scale (HAM-A), Binge Eating Scale (BES), Barratt Impulsiveness Scale (BIS-11). BIS-11 is further divided into factors, including Attention (BIS-11, A), Motor impulsiveness (BIS-11, Im), Odds ratio (OR), Self-control (BIS-11, Ac), Cognitive complexity (BIS-11, Cc), Perseverance (BIS-11, P) , Cognitive Instability (BIS-11, IC), uncorrected p-values (p_unc_).

|  | **Quantity** | | **Quality** | | **Snack** | | **Gorge** | | **Binge** | | **Emotional Eating** | |
| --- | --- | --- | --- | --- | --- | --- | --- | --- | --- | --- | --- | --- |
|  | **OR**  **95% CI** | **p_unc_** | **OR**  **95% CI** | **p_unc_** | **OR**  **95% CI** | **p_unc_** | **OR**  **95% CI** | **p_unc_** | **OR**  **95% CI** | **p_unc_** | **OR**  **95% CI** | **p_unc_** |
| **Gender** | 4.82  [1.05, 22.10] | **0.04** | 0.42  [0.14, 1.23] | 0.11 | 0.37  [0.12, 1.14] | 0.08 | 1.84  [0.63, 5.42] | 0.27 | 2.07  [0.26, 16.31] | 0.49 | 0.24  [0.08, 0.75] | **0.01** |
| **BES** | 1.08  [0.99, 1.17] | 0.07 | 1.04  [0.97, 1.12] | 0.28 | 1.06  [0.99, 1.14] | 0.11 | 1.04  [0.97, 1.12] | 0.26 | 1.19  [1.02, 1.37] | **0.02** | 1.05  [0.97, 1.14] | 0.24 |
| **BIS11-A** | 1.22  [0.88, 1.69] | 0.22 | 0.85  [0.67, 1.10] | 0.20 | 1.06  [0.80, 1.41] | 0.67 | 0.98  [0.76, 1.26] | 0.87 | 1.10  [0.72, 1.68] | 0.66 | 1.06  [0.81, 1.40] | 0.66 |
| **BIS11-Im** | 0.98  [0.85, 1.16] | 0.76 | 0.96  [0.85, 1.10] | 0.56 | 0.98  [0.86, 1.13] | 0.82 | 1.05  [0.88, 1.24] | 0.59 | 1.01  [0.70, 1.46] | 0.95 | 1.03  [0.90, 1.18] | 0.66 |
| **BIS11-Ac** | 1.09  [0.84, 1.40] | 0.51 | 1.00  [0.83, 1.20] | 0.98 | 1.03  [0.83, 1.26] | 0.82 | 1.24  [1.00, 1.54] | **0.05** | 1.29  [0.92, 1.81] | 0.14 | 1.06  [0.86, 1.30] | 0.60 |
| **BIS11-Cc** | 0.99  [0.79, 1.25] | 0.95 | 1.16  [0.92, 1.47] | 0.20 | 1.12  [0.87, 1.44] | 0.37 | 0.86  [0.69, 1.08] | 0.19 | 0.80  [0.58, 1.11] | 0.18 | 1.08  [0.85, 1.36] | 0.55 |
| **BIS11-P** | 1.02  [0.78, 1.35] | 0.87 | 0.98  [0.74, 1.28] | 0.85 | 0.83  [0.61, 1.12] | 0.22 | 1.15  [0.88, 1.52] | 0.30 | 0.76  [0.43, 1.35] | 0.32 | 0.82  [0.59, 1.13] | 0.22 |
| **BIS11-Ic** | 0.81  [0.58, 1.13] | 0.21 | 1.00  [0.76, 1.34] | 0.97 | 1.07  [0.81, 1.42] | 0.64 | 0.93  [0.69, 1.25] | 0.63 | 1.35  [0.77, 2.36] | 0.29 | 1.03  [0.74, 1.42] | 0.87 |
| **ECR-Av** | 0.99  [0.94, 1.03] | 0.56 | 0.99  [0.96, 1.03] | 0.59 | 0.97  [0.94, 1.00] | 0.08 | 1.01  [0.98, 1.04] | 0.51 | 1.05  [0.98, 1.11] | 0.15 | 1.00  [0.96, 1.05] | 0.85 |
| **ECR-An** | 1.02  [0.98, 1.06] | 0.38 | 1.02  [0.99, 1.06] | 0.26 | 0.99  [0.96, 1.02] | 0.32 | 0.98  [0.95, 1.01] | 0.24 | 0.99  [0.92, 1.07] | 0.75 | 1.02  [0.98. 1.06] | 0.46 |
| **DERS** | 0.98  [0.94, 1.02] | 0.37 | 0.99  [0.96, 1.03] | 0.74 | 1.02  [0.98, 1.06] | 0.40 | 1.01  [0.98, 1.05] | 0.51 | 0.99  [0.92, 1.07] | 0.85 | 0.97  [0.93, 1.02] | 0.20 |
| **HAM-D** | 0.99  [0.85, 1.16] | 0.90 | 1.00  [0.88, 1.13] | 0.94 | 0.90  [0.80, 1.02] | 0.09 | 1.00  [0.88, 1.13] | 0.94 | 1.00  [0.80, 1.24] | 0.97 | 1.05  [0.91, 1.19] | 0.52 |
| **HAM-A** | 0.96  [0.83, 1.11] | 0.57 | 1.00  [0.88, 1.13] | 0.99 | 1.05  [0.94, 1.19] | 0.39 | 0.97  [0.85, 1.09] | 0.57 | 1.10  [0.90, 1.35] | 0.35 | 1.11  [0.96, 1.30] | 0.16 |

**Supplementary references**

1. Gratz KL, Roemer L. Multidimensional Assessment of Emotion Regulation and Dysregulation: Development, Factor Structure, and Initial Validation of the Difficulties in Emotion Regulation Scale. *Journal of Psychopathology and Behavioral Assessment* (2004) 26:41–54. doi: 10.1023/B:JOBA.0000007455.08539.94

2. Hamilton M. A RATING SCALE FOR DEPRESSION. *Journal of Neurology, Neurosurgery & Psychiatry* (1960) 23:56–62. doi: 10.1136/jnnp.23.1.56

3. Hamilton M. THE ASSESSMENT OF ANXIETY STATES BY RATING. *British Journal of Medical Psychology* (1959) 32:50–55. doi: 10.1111/j.2044-8341.1959.tb00467.x

4. Patton JH, Stanford MS, Barratt ES. Factor structure of the barratt impulsiveness scale. *J Clin Psychol* (1995) 51:768–774. doi: 10.1002/1097-4679(199511)51:6<768::AID-JCLP2270510607>3.0.CO;2-1

5. Gormally J, Black S, Daston S, Rardin D. The assessment of binge eating severity among obese persons. *Addictive Behaviors* (1982) 7:47–55. doi: 10.1016/0306-4603(82)90024-7

6. Fraley RC, Waller NG, Brennan KA. An item response theory analysis of self-report measures of adult attachment. *Journal of Personality and Social Psychology* (2000) 78:350–365. doi: 10.1037/0022-3514.78.2.350

7. Ricca V, Castellini G, Lo Sauro C, Ravaldi C, Lapi F, Mannucci E, Rotella CM, Faravelli C. Correlations between binge eating and emotional eating in a sample of overweight subjects. *Appetite* (2009) 53:418–421. doi: 10.1016/j.appet.2009.07.008

**
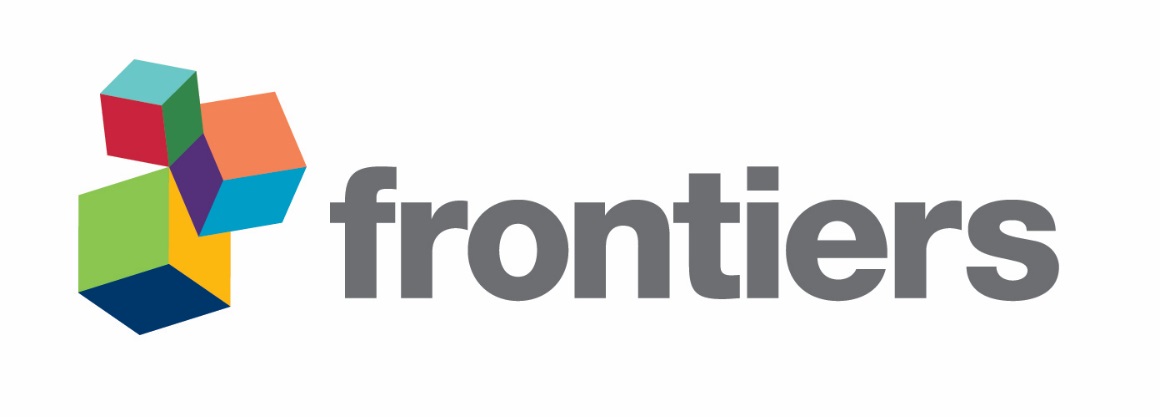
**
